# Knee Extensor Strength Is Associated with Pressure Pain Thresholds in Adults with Fibromyalgia

**DOI:** 10.1371/journal.pone.0059930

**Published:** 2013-04-02

**Authors:** W. Michael Hooten, Casandra J. Rosenberg, Jason S. Eldrige, Wenchun Qu

**Affiliations:** 1 Department of Anesthesiology, Mayo Clinic, Rochester, Minnesota, United States of America; 2 Department of Physical Medicine and Rehabilitation, Mayo Clinic, Rochester, Minnesota, United States of America; The University of Queensland, Australia

## Abstract

**Objective:**

Individuals with fibromyalgia (FM) have lower muscle strength and lower pressure pain thresholds (PPT). The primary aim of this study was to determine the associations between muscle strength and PPT in adults with FM to test the hypothesis that greater measures of muscle strength would be associated with greater values of PPT. Secondary aims included determining the effects of pain severity and the peak uptake of oxygen (Vo_2_) on the associations between muscle strength and PPT.

**Methods:**

Knee extensor and flexor strength (N = 69) was measured in the dominant leg using a dynamometer, and PPT was assessed using an electronic algometer. Pain severity was determined using the Multidimensional Pain Inventory, and peak Vo_2_ uptake was quantified using an electronically braked cycle ergometer.

**Results:**

Univariable linear regression analysis demonstrated a significant association between PPT (dependent variable) and isometric knee extensor (*P*<.001), isokinetic (60°/s) knee extensor (*P* = .002), and isokinetic (60°/s) knee flexor strength (*P* = .043). In a multiple variable linear regression analysis adjusted for age, sex, pain severity, body mass index and peak Vo_2_ uptake, a significant association was found between PPT and isometric knee extensor strength (*P* = .008). In a similar multiple variable analysis, a significant association was found between PPT and isokinetic knee extensor strength (*P* = .044).

**Conclusion:**

Greater measures of isometric and isokinetic knee extensor strength were significantly associated with greater values of PPT in both univariable and multiple variable linear regression models.

**Trial Registration:**

ClinicalTrials.gov NCT01253395

## Introduction

Individuals with fibromyalgia (FM) have been recognized to have lower knee extensor and flexor strength [1], [2], [3], and lower pressure pain thresholds (PPT) [4], [5], [6]. In previous experimental studies, patients with FM have been observed to have lower PPT during and immediately following muscle contraction [7], [8], [9]. However, the associations between measures of muscle strength and values of PPT obtained independent of active strength testing have not been fully investigated. Fibromyalgia is also associated with widespread pain [10] and lower levels of aerobic conditioning [11], [12], both of which could potentially influence the associations between muscle strength and PPT [2], [13], [14].

In a previous randomized equivalence trial, measures of knee extensor and flexor strength, PPT, peak uptake of oxygen (Vo_2_), and pain severity were assessed in a cohort of adults with FM undergoing interdisciplinary pain treatment [15]. In this previous study, maximum voluntary isometric knee extensor, isokinetic knee extensor and isokinetic knee flexor strength were assessed using a dynamometer which provided measures of maximum voluntary strength for both the hamstring and quadriceps muscle groups. Although endurance plays an important role in muscle performance, differences in muscle endurance among adults with FM compared to controls have not been consistently reported [16], [17], [18], [19]. However, dynamometric measures of maximum voluntary knee extensor and flexor strength have been widely used to investigate alterations in muscle strength among adults with FM despite the potential confounding effects of motivation and pain perception [3], [20], [21], [22]. Therefore, the primary aim of this study was to determine the associations between isometric knee extensor, isokinetic knee extensor, and isokinetic knee flexor strength and PPT in adults with FM to test the hypothesis that greater measures of muscle strength would be associated with greater values of PPT. A secondary aim was to determine the influence of pain severity, as measured by the Multidimensional Pain Inventory, and peak Vo_2_ uptake on the associations between muscle strength and PPT. The current study represents a secondary analysis of previously published data [15].

## Methods

### Study Participants

The study was approved by the Mayo Foundation Institutional Review Board, and written informed consent was obtained from all study participants. All patients were recruited at admission to the 3-week outpatient interdisciplinary pain treatment program from April 2006 through February 2008. Upon admission to the interdisciplinary pain treatment program and prior to study recruitment, all patients were fully ambulatory and able to independently maintain all activities of daily living. Inclusion criteria included an established diagnosis of fibromyalgia according to the American College of Rheumatology (ACR) 1990 diagnostic criteria [23] and age greater than 18 years. Exclusion criteria included cardiovascular (e.g., ischemic heart disease, cardiomyopathy), pulmonary (e.g., chronic obstructive pulmonary disease), orthopedic (e.g., severe osteoarthritis) or other systemic disease (e.g., rheumatoid arthritis, lupus) that could limit the assessment of knee extensor or flexor strength. Other exclusion criteria included pregnancy, and a diagnosis of schizophrenia, schizoaffective disorder, or dementia. Informed consent was obtained on the day of admission, strength testing and PPT were assessed on day 1 following admission, and peak Vo_2_ uptake was assessed on day 2 following admission. During the study period, 72 patients were recruited and 69 completed the baseline assessment.

### Demographics and Clinical Characteristics

Baseline demographic and clinical characteristics were collected at admission including age, gender, ethnicity, body mass index (kg/m^2^), employment status, pain duration, marital status, years of education, and prescription opioid use.

### Determination of Morphine Equivalent Dose

At admission, the daily opioid dose of each study subject was determined by self-report and review of pharmacy records, as previously described [24], [25]. The daily opioid dose was converted to daily morphine equivalents using an equianalgesic conversion software program [26] that has been used extensively at our outpatient treatment center [27], [28], [29].

### Pressure Pain Threshold

Musculoskeletal tender points at 18 sites were identified according to ACR criteria [23] on day 1 after admission in a purpose-designated examination room located at the interdisciplinary pain treatment center. The method for assessing PPT has been previously described [15]. In brief, PPT at all 18 specified sites were measured by the same investigator (WMH) using an electronic algometer (JTECH Medical, Salt Lake City, UT, U.S.A.) fitted with a 1.0 cm diameter footplate. Pressure was applied at a constant rate of 1 kg/sec. Study participants were instructed to indicate when the pressure sensation became painful. Each site was tested 3 times in immediate succession, and the mean pressure pain threshold of each site was calculated using software designed specifically for the algometer. The mean pressure pain threshold of the 18 sites was then calculated for each patient. The interrater and intrarater reliability of this particular algometer has been shown to be 0.89 and 0.92, respectively [30]. This method of assessing pressure pain thresholds is similar to that reported by Harden et al [4].

### Muscle Strength

All study participants underwent knee strength testing on day 1 after admission in a test session that was independent of the assessment of PPT. Strength testing was conducted in a purpose-designated biomechanics laboratory, as previously described [15]. Isometric knee extensor strength, isokinetic knee extensor strength, and isokinetic knee flexor strength were measured in the dominant leg of each subject using a dynamometer (Biodex Medical Systems, Shirley, NY, U.S.A.). This particular device has been shown to provide a reliable and valid measure of isometric and isokinetic strength [31], [32]. Subjects were seated with a hip joint angle between 90 and 100 degrees of flexion and stabilizing straps were placed over the chest, hips and thighs [33]. The knee joint was aligned with the axis of the lever arm and the leg was attached to the lever arm just above the ankle. Each subject was familiarized with the test protocol, and a brief warm-up session was conducted prior to testing. Isokinetic strength testing was performed at a speed of 60 degrees per second (60°/s) from a starting point of 90° of knee flexion. Subjects were instructed to perform maximal force voluntary contraction of knee extensors followed immediately by contraction of knee flexors in a continuous manner. For the isometric test, the lower leg position was fixed at 60° of knee flexion and subjects were instructed to perform a maximal force voluntary contraction of the knee extensors. A series of five consecutive contractions was performed for each strength measure with an intervening 1 minute rest period between series. The maximum torque production recorded from the five attempts was used for data analysis, and gravity correction was performed.

### Peak Uptake of Oxygen (Vo_2_)

All study subjects had testing of peak aerobic capacity in a purpose-designated human physiology laboratory under the direct supervision of a physician on day 2 following admission. Oxygen uptake was measured using an incremental protocol on an electronically braked cycle ergometer [33]. After an initial 3-min stage at 25 to 50 W, the workload was increased by 10 to 25 W every minute until volitional fatigue was achieved. All subjects were encouraged by the research staff throughout the test session to give maximal effort. Peak Vo_2_ uptake was defined as the greatest oxygen uptake recorded during the last 30 s of exercise. Continuous monitoring of 12-lead electrocardiogram and blood pressure were performed, and expired gases were analyzed with a mass spectrometer (Perkin Elmer, Walthman, MA). The peak Vo_2_ uptake per kilogram was used for data analysis.

### Pain Severity

Pain severity was assessed on the day of admission using the pain severity subscale of the Multidimensional Pain Inventory (MPI) [34]. This self-report questionnaire has proven reliability and construct validity [35]. The pain severity subscale is a composite measure of pain calculated from the responses to the following three questions: 1) “Rate the level of your pain at the present moment;” 2) “On average, how severe has your pain been during the last week;” and 3) “How much suffering do you experience because of your pain.” Responses to each question are scored on a 7-point Likert scale where 0 indicates no pain or suffering, and 6 indicates extreme pain or suffering. Raw scores from the pain severity subscale were converted to standardized *T*-scores using the means and standard deviations from a group of over 700 patients with chronic pain [36]. The standardized subscale has a mean of 50 (range 0 to 100) and a standard deviation (SD) of 10, where higher scores indicate greater pain severity. This standardized measure of clinical pain has been used previously to assess the outcomes of patients with fibromyalgia admitted to our pain treatment program [28], [29].

### Data Analyses

Demographics (age, sex, ethnicity, marital status, educational status, employment status) and clinical characteristics (pain duration, pain severity, frequency of opioid use, daily morphine equivalent dose) were summarized. Mean and standard deviation (SD) were reported for continuous variables, and count and proportion were reported for categorical variables.

Univariable linear regression analysis was performed with PPT as the dependent variable and measures of muscle strength (isometric knee extensor, isokinetic knee extensor, isokinetic knee flexor) as the independent variables. Multiple variable linear regression analyses were then performed and adjusted for age, sex, pain severity, BMI and peak Vo_2_ uptake. These demographic and clinical characteristics were included as cofactors because they have been previously associated with alterations in pain perception, physical functioning, and pain severity in patients with FM [2], [12], [13], and in patients admitted to our interdisciplinary treatment program [28], [29], [37], [38], [39]. The level of significance for all statistical tests was set at *P*<.05, and all analyses were completed using PAWS (IBM, Inc., Chicago, Il, Version 18.0).

## Results

### Demographic and Clinical Characteristics

Demographic and clinical characteristics have been summarized in Table 1. The majority of study participants were Caucasian females who were married and not currently employed. The average pain duration exceeded 12 years, and 28% of the group was using prescription opioids. The morphine equivalent dose ranged from 5 mg/day to 442 mg/day.

**Table 1:** Demographic and clinical characteristics.

Characteristic	(N = 69)
Sex (*N, %)*	
Female	63 (91.3)
Male	6 (8.7)
Age (*M ± SD*)	46.7±10.5
Ethnicity (*N, %)*	
Caucasian	66 (95.7)
African American	1 (1.4)
Hispanic	1 (1.4)
Other	1 (1.4)
Body mass index (kg/m^2^) (*M ± SD*)	30.2 (6.8)
Married (*N, %)*	46 (66.7)
Currently employed (*N, %)*	21 (30.4)
Frequency of opioid use (*N, %)*	19 (27.5)
Years of education (*M ± SD*)	14.9±3.2
Pain duration, years (*M ± SD*)	12.2±12.9
Morphine equivalent dose (*mg/day*)	89.5±132.2

### Associations between PPT and Muscle Strength

**Figure 1:**
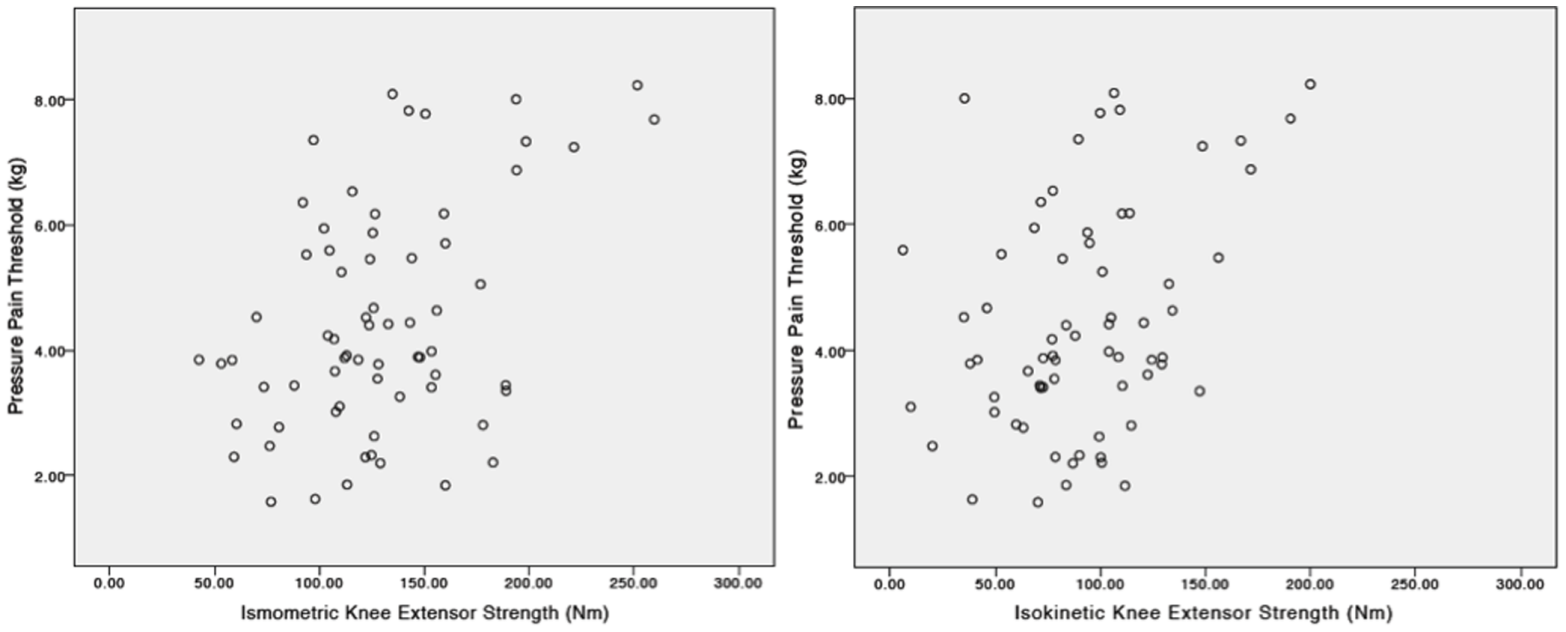
Knee Extensor Strength and Pressure Pain Threshold. This diagram demonstrates the associations between isometric knee extensor strength, isokinetic knee extensor strength and pressure pain threshold.

Table 2 contains the mean values for muscle strength, PPT, pain severity, and peak Vo_2_ uptake. In univariable linear regression analyses, a significant association was found between PPT (dependent variable) and isometric knee extensor (*P*<.001), isokinetic knee extensor (*P* = .002), and isokinetic knee flexor strength (*P* = .043) (Table 3). Figure 1 displays a graph of the significant associations between PPT and isometric knee extensor, and isokinetic knee extensor strength.

**Table 2:** Mean values of pressure pain threshold, muscle strength, peak Vo_2_ uptake, and pain severity.

Variable	(N = 69)
Pressure pain threshold (kg)	4.5±1.8
Isometric knee extensor (Nm)	130.7±45.0
Isokinetic knee extensor 60°/s (Nm)	92.9±40.3
Isokinetic knee flexor 60°/s (Nm)	53.7±23.0
Pain severity*	47.7±8.2
Peak Vo_2_ uptake (ml/kg/min)	16.5±4.2

* pain severity subscale of the Multidimensional Pain Inventor.

**Table 3:** Linear regression analyses with pressure pain threshold as the dependent variable.

Variable	B coefficient univariableanalysis (95% CI)	*P* Value	B coefficient multiple variableanalysis (95% CI)*	*P* Value
Isometric knee extensor	.018 (.009 to.027)	<.001	.015 (.004 to.026)	.008
Isokinetic knee extensor 60°/s	.017 (.006 to.027)	.002	.012 (.000 to.024)	.044
Isokinetic knee flexor 60°/s	.020 (.001 to.039)	.043	.006 (−.016 to.028)	.577

* adjusted for age, sex, pain severity, BMI and peak Vo_2_ uptake.

In a multiple variable linear regression analysis adjusted for age, sex, pain severity, BMI and peak Vo_2_ uptake, a significant association was found between PPT (dependent variable) and isometric knee extensor strength (independent variable) (*P* = .008), where every one point (Nm) increase in isometric knee extensor strength was associated with an 15 g increase in PPT (Table 3). A significant factor in the regression model included pain severity (B coefficient = –.061; *P* = .016), where every one point increase in pain severity was associated with an approximate 6 g decrease in PPT.

In a similar multiple variable linear regression analysis adjusted for age, sex, pain severity, BMI and peak Vo_2_ uptake, a significant association was found between PPT (dependent variable) and isokinetic knee extensor strength (independent variable) (*P* = .044), where every one point (Nm) increase in extensor strength was associated with a 12 g increase in PPT (Table 3). Other significant factors in this regression model included pain severity (B coefficient = –.058; *P* = .027) and sex (B coefficient = −1.7, *P* = .023) where greater pain severity and female sex were associated with a lower PPT.

In a multiple variable linear regression analysis adjusted for age, sex, pain severity, BMI, and peak Vo_2_ uptake, no significant association was found between PPT (dependent variable) and isokinetic knee flexor strength (independent variable) (*P* = .577). Significant factors in the regression model included pain severity (B coefficient = –.063; *P* = .020) and sex (B coefficient = −1.8, *P* = .024), where greater pain severity and female sex were associated with lower PPT.

## Discussion

The main findings of this study were that greater measures of isometric and isokinetic knee extensor strength were significantly associated with greater values of PPT in both univariable and multiple variable regression models adjusted for age, sex, opioid use, pain severity, BMI, and peak Vo_2_ uptake. In all multiple variable regression models, greater pain severity scores were associated with lower values of PPT, but no significant associations were observed between peak Vo_2_ uptake and PPT.

Previous studies have consistently found lower values of PPT, assessed using an algometer, during and up to 5 minutes following assessment of isometric knee extensor strength in patients with FM compared to healthy controls [7], [8], [9]. More specifically, lower values of PPT have been observed over the quadriceps and resting homologous contralateral muscle groups [40], [41], [42]. Dysfunction in pain inhibitory and activation of pain facilitatory mechanisms have been posited to explain, in part, lower values of PPT obtained over painful and distant non-painful muscle groups, respectively [43]. The methods used in our study were distinct from those used in previous investigations. First, the values of PPT were obtained independent of strength testing. Second, the mean PPT of the 18 ACR tender point sites [23] was used to assess pain perception compared to PPT obtained over a single muscle group. Using different methods, our findings extend the observations of previous studies and suggest that greater measures of muscle strength could influence the peripheral mechanisms implicated in the maintenance of central sensitization in patients with FM [44], [45]. For example, various metabolic factors including alterations in ATP, phosphocreatine, and nitric oxide levels could lead to alterations in tissue oxygenation, and contribute to the development and maintenance of peripheral sensitization [46], [47], [48]. However, the specific mechanisms by which greater muscle strength could effect peripheral and central sensitization have not been elucidated.

Greater pain severity was associated with lower PPT in all multiple variable models. This is consistent with previous studies where greater measures of clinical pain have been associated with lower PPT and greater tender point counts [49], [50]. Furthermore, the occurrence of pain impairs muscle function due, in part, to the input of joint nociceptors on motor neurons which can reduce the capacity to activate and coordinate muscle contractions [51], [52], [53]. More recent findings also suggest that motor unit recruitment can be independently altered by descending inputs related to the cognitions of pain anticipation without concomitant nociceptive discharge [54]. The observations from our study suggest that the influence of muscle strength on PPT occurred independent of pain severity, which further supports the supposition that muscle strength could play an important role in peripheral sensitization among individuals with FM. However, peak Vo_2_ uptake was not associated with PPT. Although an association between peak Vo_2_ uptake and tender point counts has been reported in some, but not all studies [12], [20], [55], [56], the effect of peak Vo_2_ uptake on PPT has not been widely investigated. The findings from this study suggest that peak Vo_2_ uptake does not have a direct effect on peripheral sensitization in FM.

The findings from this study have potential implications for the use of isometric and isokinetic knee extensor strength in ongoing clinical research and practice. First, greater isometric and isokinetic knee extensor strength was associated with lower values of PPT at a single time point in the multiple variable regression models, but further research is needed to determine if immediate changes in these measures of knee extensor strength following strength training would be predictive of greater values of PPT. Although it is not entirely clear why knee extensor but not knee flexor strength was associated with lower values of PPT, previous investigators have postulated that the greater torque generating capacity of knee extension and nociceptive input from the patello-femoral joint may be contributing factors [57], [58]. Second, further research is also needed to determine if repeated assessments of isometric and isokinetic knee extensor strength over time would be a useful indicator of sustained improvements in PPT among patients engaged in maintenance strength training programs. If these lines of research prove successful, the assessment of isometric and isokinetic knee extensor strength could be a useful, noninvasive tool for investigating the immediate and long-term effects of various strength training interventions on PPT in adults with FM.

The study has limitations. Study participants were specifically referred for interdisciplinary pain treatment at a tertiary medical center, and the majority of participants were Caucasian women. Therefore, referral bias may limit the generalization of the study findings to other populations of adults with FM. However, the clinical characteristics of patients admitted to the pain program were comparable to a random sample of community pain patients from the catchment area of our medical center [59]. Similarly, the study was conducted exclusively at a specialized outpatient pain treatment center which could limit the generalization of the study observations to other ambulatory care settings. Although patients recruited for study participation were screened for underlying medical and surgical conditions that could limit the assessment of knee extensor and flexor strength, the assessment of maximal voluntary strength would not be expected to identify individual patients who may have had unrecognized muscle dysfunction due, in part, to reduced muscle mass or abnormal strength production capacity. However, the mean maximal voluntary strength values of our cohort were comparable to mean normative values for women age 30 to 50 years where isometric knee extensor, isokinetic knee extensor (60°/s) and isokinetic knee flexor (60°/s) strength have been reported to range from 123–147 Nm, 99–113 Nm, and 66–93 Nm, respectively [60], [61], [62], [63]. Although peak Vo_2_ uptake was assessed using an incremental protocol on an electronically braked cycle ergometer, other physiological measures of peak aerobic capacity, including maximal heart rate, were not correlated with muscle strength, and serum lactate levels were not measured. The mean PPT of the 18 FM tender point sites was used rather than a measure of PPT derived from the knee or other lower extremity region. The PPT of the 18 FM tender point sites was used because FM is clinically characterized by widespread reductions in PPT as opposed to lower PPT localized to any one specific body region. Therefore, the PPT from the 18 FM tender point sites should be a more representative measure of the widespread changes in PPT that occur in patients with FM. Lastly, the strength of the quadriceps and hamstring groups was tested, and replication of our results in other muscle groups would further verify the observed associations between muscle strength and PPT.

The findings reported herein extend the results of previous experimental studies and suggest that greater isometric and isokinetic knee extensor strength were associated with greater values of PPT in both univariable and multiple variable linear regression analyses. Ongoing research is needed to further elucidate the immediate and long-term clinical effects of strength training on values of PPT in adults with FM.

## References

[1] Goes SM, Leite N, Shay BL, Homann D, Stefanello JM, et al (2012) Functional capacity, muscle strength and falls in women with fibromyalgia. Clin Biomech (Bristol, Avon) 27: 578–583. 10.1016/j.clinbiomech.2011.12.009 22230426

[2] Bachasson D, Guinot M, Wuyam B, Favre-Juvin A, Millet GY, et al.. (2012) Neuromuscular fatigue and exercise capacity in fibromyalgia syndrome. Arthritis Care Res (Hoboken). 10.1002/acr.21845 22965792

[3] Valkeinen H, Hakkinen A, Hannonen P, Hakkinen K, Alen M (2006) Acute heavy-resistance exercise-induced pain and neuromuscular fatigue in elderly women with fibromyalgia and in healthy controls: effects of strength training. Arthritis Rheum 54: 1334–1339. 16575859 10.1002/art.21751

[4] Harden RN, Revivo G, Song S, Nampiaparampil D, Golden G, et al (2007) A critical analysis of the tender points in fibromyalgia. Pain Med 8: 147–156. 17305686 10.1111/j.1526-4637.2006.00203.x

[5] Amris K, Jespersen A, Bliddal H (2010) Self-reported somatosensory symptoms of neuropathic pain in fibromyalgia and chronic widespread pain correlate with tender point count and pressure-pain thresholds. Pain 151: 664–669. 20832941 10.1016/j.pain.2010.08.023

[6] Marques AP, Ferreira EA, Matsutani LA, Pereira CA, Assumpcao A (2005) Quantifying pain threshold and quality of life of fibromyalgia patients. Clin Rheumatol 24: 266–271. 15616761 10.1007/s10067-004-1003-7

[7] Kosek E, Ekholm J, Hansson P (1996) Sensory dysfunction in fibromyalgia patients with implications for pathogenic mechanisms. Pain 68: 375–383. 9121827 10.1016/s0304-3959(96)03188-0

[8] Kosek E, Ekholm J, Hansson P (1996) Modulation of pressure pain thresholds during and following isometric contraction in patients with fibromyalgia and in healthy controls. Pain 64: 415–423. 8783304 10.1016/0304-3959(95)00112-3

[9] Kosek E, Hansson P (1997) Modulatory influence on somatosensory perception from vibration and heterotopic noxious conditioning stimulation (HNCS) in fibromyalgia patients and healthy subjects. Pain 70: 41–51. 9106808 10.1016/s0304-3959(96)03295-2

[10] Staud R, Domingo M (2001) Evidence for abnormal pain processing in fibromyalgia syndrome. Pain Med 2: 208–215. 15102253 10.1046/j.1526-4637.2001.01030.x

[11] Dinler M, Diracoglu D, Kasikcioglu E, Sayli O, Akin A, et al (2009) Effect of aerobic exercise training on oxygen uptake and kinetics in patients with fibromyalgia. Rheumatol Int 30: 281–284. 19784655 10.1007/s00296-009-1126-x

[12] Redondo JR, Justo CM, Moraleda FV, Velayos YG, Puche JJ, et al (2004) Long-term efficacy of therapy in patients with fibromyalgia: a physical exercise-based program and a cognitive-behavioral approach. Arthritis Rheum 51: 184–192. 15077258 10.1002/art.20252

[13] Miller TA, Allen GM, Gandevia SC (1996) Muscle force, perceived effort, and voluntary activation of the elbow flexors assessed with sensitive twitch interpolation in fibromyalgia. J Rheumatol 23: 1621–1627. 8877935

[14] Murthy G, Hargens AR, Lehman S, Rempel DM (2001) Ischemia causes muscle fatigue. J Orthop Res 19: 436–440. 11398857 10.1016/S0736-0266(00)90019-6

[15] Hooten WM, Qu W, Townsend CO, Judd JW (2012) Effects of strength vs aerobic exercise on pain severity in adults with fibromyalgia: a randomized equivalence trial. Pain 153: 915–923. 22341565 10.1016/j.pain.2012.01.020

[16] Carbonell-Baeza A, Aparicio VA, Sjostrom M, Ruiz JR, Delgado-Fernandez M (2011) Pain and functional capacity in female fibromyalgia patients. Pain Med 12: 1667–1675. 21939495 10.1111/j.1526-4637.2011.01239.x

[17] Jacobsen S, Wildschiodtz G, Danneskiold-Samsoe B (1991) Isokinetic and isometric muscle strength combined with transcutaneous electrical muscle stimulation in primary fibromyalgia syndrome. J Rheumatol 18: 1390–1393. 1757942

[18] Maquet D, Croisier JL, Renard C, Crielaard JM (2002) Muscle performance in patients with fibromyalgia. Joint Bone Spine 69: 293–299. 12102276 10.1016/s1297-319x(02)00373-1

[19] Norregaard J, Bulow PM, Danneskiold-Samsoe B (1994) Muscle strength, voluntary activation, twitch properties, and endurance in patients with fibromyalgia. J Neurol Neurosurg Psychiatry 57: 1106–1111. 8089679 10.1136/jnnp.57.9.1106 PMC1073137

[20] Gowans SE, deHueck A, Voss S, Silaj A, Abbey SE, et al (2001) Effect of a randomized, controlled trial of exercise on mood and physical function in individuals with fibromyalgia. Arthritis Rheum 45: 519–529. 11762686 10.1002/1529-0131(200112)45:6<519::aid-art377>3.0.co;2-3

[21] Tomas-Carus P, Gusi N, Hakkinen A, Hakkinen K, Raimundo A, et al (2009) Improvements of muscle strength predicted benefits in HRQOL and postural balance in women with fibromyalgia: an 8-month randomized controlled trial. Rheumatology (Oxford) 48: 1147–1151. 19605373 10.1093/rheumatology/kep208

[22] Valkeinen H, Alen M, Hannonen P, Hakkinen A, Airaksinen O, et al (2004) Changes in knee extension and flexor force, EMG and functional capacity during strength training in older females with fibromyalgia and healthy controls. Rheumatology (Oxford) 43: 225–228. 13130154 10.1093/rheumatology/keh027

[23] Wolfe F, Smythe HA, Yunus MB, Bennett RM, Bombardier C, et al (1990) The American College of Rheumatology 1990 Criteria for the Classification of Fibromyalgia. Report of the Multicenter Criteria Committee. Arthritis Rheum 33: 160–172. 2306288 10.1002/art.1780330203

[24] Cunningham JL, Rome JD, Kerkvliet JL, Townsend CO (2009) Reduction in medication costs for patients with chronic nonmalignant pain completing a pain rehabilitation program: a prospective analysis of admission, discharge, and 6-month follow-up medication costs. Pain Med 10: 787–796. 19302437 10.1111/j.1526-4637.2009.00582.x

[25] Townsend CO, Kerkvliet JL, Bruce BK, Rome JD, Michael Hooten W, et al (2008) A longitudinal study of the efficacy of a comprehensive pain rehabilitation program with opioid withdrawal: Comparison of treatment outcomes based on opioid use status at admission. Pain 140: 177–189. 18804915 10.1016/j.pain.2008.08.005

[26] DuPen S, DuPen A (2000) Opioid conversion calculator. Poulsbo, WA: Cynergy Group.

[27] Cunningham JL, Rome JD, Kerkvliet JL, Townsend CO (2009) Reduction in medication costs for patients with chronic nonmalignant pain completing a pain rehabilitation program: a prospective analysis of admission, discharge, and 6-month follow-up medication costs. Pain medicine 10: 787–796. 19302437 10.1111/j.1526-4637.2009.00582.x

[28] Hooten WM, Townsend CO, Decker PA (2007) Gender differences among patients with fibromyalgia undergoing multidisciplinary pain rehabilitation. Pain medicine 8: 624–632. 18028040 10.1111/j.1526-4637.2006.00202.x

[29] Hooten WM, Townsend CO, Sletten CD, Bruce BK, Rome JD (2007) Treatment outcomes after multidisciplinary pain rehabilitation with analgesic medication withdrawal for patients with fibromyalgia. Pain medicine 8: 8–16. 17244099 10.1111/j.1526-4637.2007.00253.x

[30] Liu F, Carlson L, Watson HK (2000) Quantitative abductor pollicis brevis strength testing: reliability and normative values. J Hand Surg Am 25: 752–759. 10913219 10.1053/jhsu.2000.6462

[31] Drouin JM, Valovich-mcLeod TC, Shultz SJ, Gansneder BM, Perrin DH (2004) Reliability and validity of the Biodex system 3 pro isokinetic dynamometer velocity, torque and position measurements. Eur J Appl Physiol 91: 22–29. 14508689 10.1007/s00421-003-0933-0

[32] Lund H, Sondergaard K, Zachariassen T, Christensen R, Bulow P, et al (2005) Learning effect of isokinetic measurements in healthy subjects, and reliability and comparability of Biodex and Lido dynamometers. Clin Physiol Funct Imaging 25: 75–82. 15725305 10.1111/j.1475-097X.2004.00593.x

[33] Short KR, Vittone JL, Bigelow ML, Proctor DN, Coenen-Schimke JM, et al (2005) Changes in myosin heavy chain mRNA and protein expression in human skeletal muscle with age and endurance exercise training. Journal of applied physiology 99: 95–102. 15746299 10.1152/japplphysiol.00129.2005

[34] Kerns RD, Turk DC, Rudy TE (1985) The West Haven-Yale Multidimensional Pain Inventory (WHYMPI). Pain 23: 345–356. 4088697 10.1016/0304-3959(85)90004-1

[35] Bernstein IH, Jaremko ME, Hinkley BS (1995) On the utility of the West Haven-Yale Multidimensional Pain Inventory. Spine 20: 956–963. 7644962 10.1097/00007632-199504150-00014

[36] Rudy TE (1989) Multiaxial Assessment of Multidimensional Pain Inventory: Computer Program User’s Manual. Pittsburgh, PA: University of Pittsburgh.

[37] Darchuk KM, Townsend CO, Rome JD, Bruce BK, Hooten WM (2010) Longitudinal treatment outcomes for geriatric patients with chronic non-cancer pain at an interdisciplinary pain rehabilitation program. Pain Med 11: 1352–1364. 20735746 10.1111/j.1526-4637.2010.00937.x

[38] Hooten WM, Mantilla CB, Sandroni P, Townsend CO (2010) Associations between heat pain perception and opioid dose among patients with chronic pain undergoing opioid tapering. Pain Med 11: 1587–1598. 21029354 10.1111/j.1526-4637.2010.00962.x

[39] Hooten WM, Sandroni P, Mantilla CB, Townsend CO (2010) Associations between heat pain perception and pain severity among patients with chronic pain. Pain Med 11: 1554–1563. 20807344 10.1111/j.1526-4637.2010.00940.x

[40] Jespersen A, Dreyer L, Kendall S, Graven-Nielsen T, Arendt-Nielsen L, et al (2007) Computerized cuff pressure algometry: A new method to assess deep-tissue hypersensitivity in fibromyalgia. Pain 131: 57–62. 17257757 10.1016/j.pain.2006.12.012

[41] Kadetoff D, Kosek E (2007) The effects of static muscular contraction on blood pressure, heart rate, pain ratings and pressure pain thresholds in healthy individuals and patients with fibromyalgia. Eur J Pain 11: 39–47. 16480906 10.1016/j.ejpain.2005.12.013

[42] Kosek E, Lundberg L (2003) Segmental and plurisegmental modulation of pressure pain thresholds during static muscle contractions in healthy individuals. Eur J Pain 7: 251–258. 12725848 10.1016/S1090-3801(02)00124-6

[43] Lannersten L, Kosek E (2010) Dysfunction of endogenous pain inhibition during exercise with painful muscles in patients with shoulder myalgia and fibromyalgia. Pain 151: 77–86. 20621420 10.1016/j.pain.2010.06.021

[44] Staud R, Nagel S, Robinson ME, Price DD (2009) Enhanced central pain processing of fibromyalgia patients is maintained by muscle afferent input: a randomized, double-blind, placebo-controlled study. Pain 145: 96–104. 19540671 10.1016/j.pain.2009.05.020 PMC2751583

[45] Staud R, Robinson ME, Weyl EE, Price DD (2010) Pain variability in fibromyalgia is related to activity and rest: role of peripheral tissue impulse input. The journal of pain : official journal of the American Pain Society 11: 1376–1383. 20451465 10.1016/j.jpain.2010.03.011 PMC2932794

[46] McIver KL, Evans C, Kraus RM, Ispas L, Sciotti VM, et al (2006) NO-mediated alterations in skeletal muscle nutritive blood flow and lactate metabolism in fibromyalgia. Pain 120: 161–169. 16376018 10.1016/j.pain.2005.10.032

[47] Park JH, Phothimat P, Oates CT, Hernanz-Schulman M, Olsen NJ (1998) Use of P-31 magnetic resonance spectroscopy to detect metabolic abnormalities in muscles of patients with fibromyalgia. Arthritis and rheumatism 41: 406–413. 9506567 10.1002/1529-0131(199803)41:3<406::AID-ART5>3.0.CO;2-L

[48] Sprott H, Rzanny R, Reichenbach JR, Kaiser WA, Hein G, et al (2000) 31P magnetic resonance spectroscopy in fibromyalgic muscle. Rheumatology (Oxford) 39: 1121–1125. 11035133 10.1093/rheumatology/39.10.1121

[49] Tastekin N, Birtane M, Uzunca K (2007) Which of the three different tender points assessment methods is more useful for predicting the severity of fibromyalgia syndrome? Rheumatol Int 27: 447–451. 17028859 10.1007/s00296-006-0232-2

[50] Salli A, Yilmaz H, Ugurlu H (2012) The relationship between tender point count and disease severity in patients with primary fibromyalgia. Rheumatol Int 32: 105–107. 20676644 10.1007/s00296-010-1580-5

[51] Hodges PW, Mellor R, Crossley K, Bennell K (2009) Pain induced by injection of hypertonic saline into the infrapatellar fat pad and effect on coordination of the quadriceps muscles. Arthritis Rheum 61: 70–77. 19116977 10.1002/art.24089

[52] Rice DA, McNair PJ (2010) Quadriceps arthrogenic muscle inhibition: neural mechanisms and treatment perspectives. Semin Arthritis Rheum 40: 250–266. 19954822 10.1016/j.semarthrit.2009.10.001

[53] Schaible HG, Grubb BD (1993) Afferent and spinal mechanisms of joint pain. Pain 55: 5–54. 8278210 10.1016/0304-3959(93)90183-P

[54] Tucker K, Larsson AK, Oknelid S, Hodges P (2012) Similar alteration of motor unit recruitment strategies during the anticipation and experience of pain. Pain 153: 636–643. 22209423 10.1016/j.pain.2011.11.024

[55] Da Costa D, Abrahamowicz M, Lowensteyn I, Bernatsky S, Dritsa M, et al (2005) A randomized clinical trial of an individualized home-based exercise programme for women with fibromyalgia. Rheumatology (Oxford) 44: 1422–1427. 16030079 10.1093/rheumatology/kei032

[56] Richards SC, Scott DL (2002) Prescribed exercise in people with fibromyalgia: parallel group randomised controlled trial. BMJ 325: 185. 12142304 10.1136/bmj.325.7357.185 PMC117444

[57] Almosnino S, Brandon SC, Sled EA (2012) Does choice of angular velocity affect pain level during isokinetic strength testing of knee osteoarthritis patients? Eur J Phys Rehabil Med 48: 569–575. 22713541

[58] Hinman RS, Crossley KM (2007) Patellofemoral joint osteoarthritis: an important subgroup of knee osteoarthritis. Rheumatology (Oxford) 46: 1057–1062. 17500072 10.1093/rheumatology/kem114

[59] Watkins EA, Wollan PC, Melton LJ, 3rd, Yawn BP (2008) A population in pain: report from the Olmsted County health study. Pain medicine 9: 166–174. 18298699 10.1111/j.1526-4637.2007.00280.x

[60] Borges O (1989) Isometric and isokinetic knee extension and flexor torque in men and women aged 20–70. Scand J Rehabil Med 21: 45–53. 2711137

[61] Fisher NM, Pendergast DR, Calkins EC (1990) Maximal isometric torque of knee extension as a function of muscle length in subjects of advancing age. Arch Phys Med Rehabil 71: 729–734. 2403277

[62] Gross MT, Credle JK, Hopkins LA, Kollins TM (1990) Validity of knee flexor and extension peak torque prediction models. Phys Ther 70: 3–10. 2294529 10.1093/ptj/70.1.3

[63] Gross MT, McGrain P, Demilio N, Plyler L (1989) Relationship between multiple predictor variables and normal knee torque production. Phys Ther 69: 54–62. 2911617 10.1093/ptj/69.1.54

